# The real-world management of acute mesenteric ischemia in Spain: results from a multicenter national survey

**DOI:** 10.1007/s00068-026-03209-1

**Published:** 2026-05-19

**Authors:** Ana-Maria González-Castillo, Laura Calsina Juscafresa, Arantxa Gelabert, Alina Velescu, Enrico Marrano, Pirkka Vikatmaa, Matti Tolonen

**Affiliations:** 1 Trauma and Emergency Surgery Unit, Section of General Surgery, Department of General Surgery, Hospital del Mar., Hospital del Mar Medical Research Institute (IMIM), Barcelona, 08003 Spain; 2 Pompeu Fabra University, Barcelona, Spain; 3 Department of Angiology Vascular and Endovascular Surgery, Hospital del Mar Medical Research Institute (IMIM), Barcelona, Spain; 4 Interventional Radiology Unit, Radiology Department, Hospital Clínic de Barcelona, University of Barcelona, Barcelona, Spain; 5 Department of Angiology Vascular and Endovascular Surgery, CIBER Vascular, Hospital del Mar Research Institute (IMIM), Barcelona, Spain; 6 Trauma and Emergency Surgery Unit, Department of General Surgery, Germans Trias I, Pujol University Hospital, Badalona, Spain; 7 Abdominal Cnter, Vascular Surgery, HUS Helsinki University Hospital, University of Helsinki, Helsinki, Finland; 8 Abdominal Center, Emergency Surgery, HUS Helsinki University Hospital, University of Helsinki, Helsinki, Finland

**Keywords:** Acute mesenteric ischemia, Mesenteric ischemia, Intestinal ischemia, Multidisciplinary organization, Practice patterns, Clinical pathways, Care bundle, Team-based care

## Abstract

**Background:**

Acute mesenteric ischemia (AMI) remains one of the most lethal vascular emergencies, with in-hospital mortality rates frequently exceeding 50%. Although early diagnosis and timely revascularization are critical, clinical practice remains highly variable. This study aimed to evaluate the real-world management of AMI in Spain, identifying gaps in resources, protocols, and interhospital coordination.

**Methods:**

A national cross-sectional survey was conducted between March and August 2024 using the Survio^®^ platform. The questionnaire was distributed to general surgeons through national surgical societies and included 27 items covering hospital infrastructure, clinical protocols, diagnostic and therapeutic availability, personal experience, and perceived system-level barriers.

**Results:**

A total of 291 surgeons responded. The median age was 40 years (IQR: 17). Most were consultants (76.3%) working in tertiary (42.6%), secondary (33%), or community hospitals (24.4%). While 97.6% reported access to 24/7 multiphasic CT and 90.4% to round-the-clock radiology, only 51.9% had 24/7 interventional radiology and 60.1% vascular surgery. Just 26.8% had institutional AMI protocols. The median distance to a referral center was 25 km (range: 2–250 km), and 68.4% reported difficulty in patient transfers. While 96.9% felt competent managing AMI, only 36.4% were familiar with the term “intestinal stroke.” A total of 76.3% expressed interest in joining a national AMI registry.

**Conclusions:**

Spanish surgeons report high self-perceived clinical competence in AMI management, but systemic fragmentation, lack of protocols, and logistical barriers limit optimal care. These findings underscore the urgent need for coordinated regional networks, standardized care pathways, and multidisciplinary collaboration to improve outcomes in acute mesenteric ischemia across European healthcare systems.

**Trial registration:**

Retrospectively registred and recorded in Clinical Trials. NCT06428240, registration date on 20th/05/2024.

**Supplementary Information:**

The online version contains supplementary material available at 10.1007/s00068-026-03209-1.

## Introduction

Acute mesenteric ischemia (AMI) represents one of the most lethal gastrointestinal emergencies, with a reported mortality rate often exceeding 50% [1–3]. The clinical presentation is nonspecific, and delays in diagnosis remain common despite advances in imaging and surgical care [4, 5]. The condition has multiple etiologies, including embolic, thrombotic, and non-occlusive mechanisms, each with different prognostic implications [5, 6].

Guidelines from the European Society for Trauma and Emergency Surgery (ESTES), the World Society of Emergency Surgery (WSES), and the European Society for Vascular Surgery (ESVS) emphasize prompt diagnosis with contrast-enhanced CT angiography and subsequent multidisciplinary management [2, 3, 7, 8]. In this context, access to hybrid operating suites has emerged as a critical resource, since they enable both, open laparotomy and endovascular revascularization in the same setting, ensuring that all therapeutic options remain available without delay. However, real-world adherence to these guidelines in daily clinical practice remains largely unknown. Recent studies by Tolonen et al. [9, 10] emphasize the impact of structured care pathways and national-level coordination in improving outcomes for AMI. These findings underscore the relevance of examining institutional variability and system-level barriers. Biphasic CT angiography has shown promise in detecting early mesenteric ischemia, with growing support for its use as a diagnostic standard [11, 12].

To date, Spain lacks a national registry and there are no published data on how AMI is managed in routine clinical practice all over the country. This fragmented approach may contribute to the persistently high mortality seen in observational studies [13, 14]. In Catalonia, a pioneering project has been launched to unify the management of AMI under a single standardized protocol. This initiative aims to streamline patient referral and clinical decision-making across the region. The protocol is being developed collaboratively by a multidisciplinary working group that brings together professionals from various scientific societies. Participation is open to all interested clinicians, fostering a shared, coordinated approach to improving AMI outcomes.

This study aimed to explore the real-world management of AMI from the perspective of Spanish surgeons through a nationwide survey, focusing on institutional protocols, resource availability, and barriers to timely care.

## Methods

We conducted a nationwide, cross-sectional study using an online survey distributed between March and August 2024. The questionnaire was designed using Survio^®^, a secure digital platform for building and collecting anonymous survey responses. The survey was sent by email to general surgeons who are members of the Spanish Association of Surgeons (Asociación Española de Cirujanos) and had agreed to receive communications via email. In total, the survey invitation was distributed to 2,900 surgeons. A total of 945 recipients opened the invitation (32.6%), and 291 completed the survey, corresponding to a response rate of 10%.

The survey included 27 items covering the following domains (Translated in Appendix 1):

- Demographics and professional role: age, position (resident, consultant, section head, department head), and hospital type (tertiary, secondary, or community).
- Diagnostic capabilities: 24/7 availability of multiphasic CT, radiology services, and angiographic imaging.
- Therapeutic resources: access to vascular surgery and interventional radiology services at any time.
- Institutional preparedness: existence of formal clinical protocols or guidelines for AMI.
- Clinical experience and knowledge: self-reported competence in managing AMI, familiarity with “intestinal stroke” terminology, experience with open abdomen techniques.
- System-level barriers: perceived difficulty in transferring patients to higher-level care facilities.
- Engagement with national initiatives: willingness to participate in a Spanish AMI registry.

Participation was anonymous and voluntary. Considering the rarity of the condition addressed in the survey, this number of responses provides a meaningful overview of current clinical practice. According to national regulations, ethics committee approval was not required for anonymous surveys without patient data. Descriptive and comparative statistical analyses were performed using SPSS. Chi-squared tests were used to evaluate associations between hospital type and categorical survey responses. Descriptive statistics were performed for all variables. Categorical variables were summarized as counts and percentages. For continuous variables, normality was assessed using the Shapiro–Wilk test. Given that the age distribution was not normal (*p* < 0.001), results were reported as median and interquartile range (IQR).

## Results

A total of 291 general surgeons completed the national survey (Appendix 1). The median age of respondents was 40 years old, with an interquartile range of 17 years. Most respondents were consultants (222;76.3%), while the rest included residents, section heads, and department chiefs. Regarding hospital type, 124 (42.6%) worked in tertiary care centers, 96 (33.0%) in secondary-level hospitals, and 71 (24.4%) in community or regional institutions (Table 1).

**Table 1:** Surgeon’s demographics and hospital characteristics

Variables		Result
Demographics	Age*	40 (17)
Professional Role (n,%)	Consultant	222 (76.3%)
	Resident	29 (10%)
	Section Chief	27 (9.2%)
	Department Head	13 (4.5%)
Hospital Level (n,%)	Tertiary Hospital	124 (42.6%)
	Secondary Hospital	96 (33%)
	Community Hospital	71 (24.4%)

*y/o (IQR)

Access to diagnostic resources was generally high: 284 (97.6%) of respondents reported having 24/7 availability of multiphasic abdominal computed tomography, and 263 (90.4%) indicated continuous access to radiologists. However, access to advanced therapeutic options was more limited. Only 151 (51.9%) of participants reported that interventional radiology was available at all times in their institution, and just 175 (60.1%) had continuous access to vascular surgery services. These disparities were more pronounced in smaller hospitals.

**Fig. 1:**
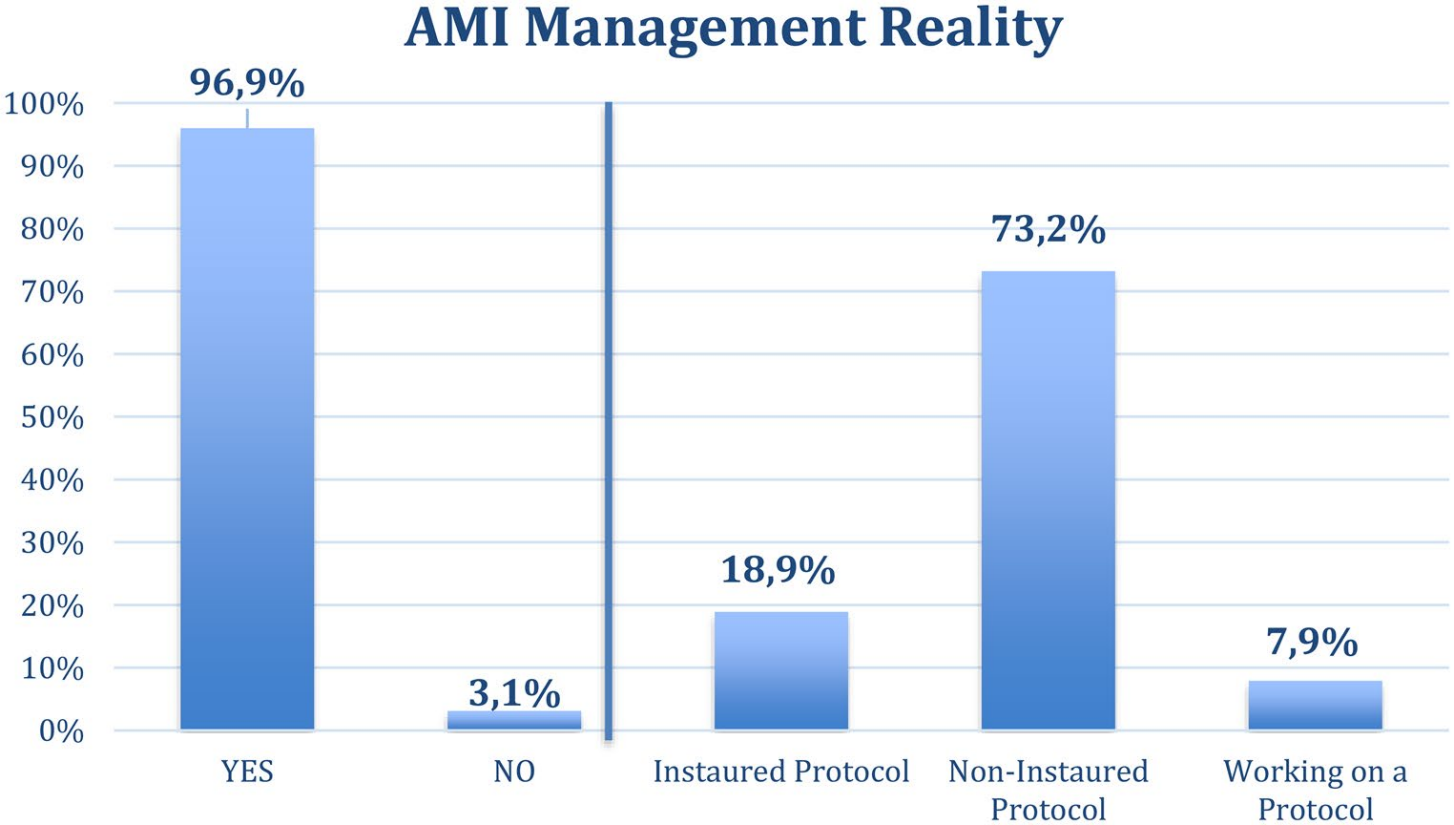
Self-reported knowledge regarding AMI and Institutional protocol. The left panel shows the response to the question “*Do you know how to manage AMI?” (YES/NO)* while the right panel shows responses to question “*Does your service have a clinical protocol or guideline for AMI?”*

In terms of institutional preparedness, only 78 (26.8%) of respondents stated that their hospital had or was working in a formal clinical protocol for managing acute mesenteric ischemia (Fig. 1). Nevertheless, the vast majority (282;96.9%) reported being familiar with the clinical management of AMI. A high percentage (279;95.9%) had personal experience with open abdomen techniques, and 115 (39.5%) had previously performed surgical revascularization of the superior mesenteric artery.

When exploring conceptual awareness, only 106 (36.4%) of surgeons were familiar with the term “intestinal stroke,” suggesting that this framing of the pathology is not yet widespread in clinical practice (Fig. 1). In contrast, systemic barriers were frequently acknowledged: 199 (68.4%) of respondents indicated that interhospital transfers for patients with AMI were excessively difficult, citing limitations in receiving capacity, delays in acceptance, and a lack of coordinated referral protocols.

One open-ended question asked respondents to estimate the distance to their nearest referral center with vascular surgery. Among those who answered, the distribution of distances was clearly non-normal. The median reported distance was 25 km, with an interquartile range of 50 km. Values ranged from as little as 2 km to as much as 250 km, revealing wide geographic disparities in access to specialized care.

A total of 184 (63.2%) respondents provided open-ended comments in the final section of the survey. Thematic analysis revealed several recurring issues. The most frequently mentioned barriers included delayed diagnosis, often attributed to low clinical suspicion or emergency department overload; lack of standardized institutional protocols; and significant challenges in coordinating interhospital transfers. Respondents also emphasized limited availability of interventional radiology and vascular surgery, particularly outside standard working hours, as well as the absence of multidisciplinary coordination in many centers. Additionally, many described high patient mortality and low individual exposure to mesenteric ischemia cases as reinforcing a negative cycle of underdiagnosis and inexperience (Table 2).

**Table 2:** Themes emerging from open-ended survey responses

Theme	Description
Delayed Diagnosis	Frequent delays in recognition, often due to low suspicion or the emergency department overloaded
Lack of standardized protocols	Absence of local or inter-hospital clinical pathways leading to inconsistent care
Difficult interhospital transfers	Time-consuming or denied transfers to tertiary hospitals
Limited 24/7 specialist availability	Non-on-call interventional radiology or vascular surgery services in many hospitals
Variability in clinical decisions	Management depends heavily on individual clinicians, especially out-of-hours
High mortality and low exposure	Rare encounters lead to limited experience, contributing to poor outcomes
Multidisciplinary coordination deficits	Lack of organized teamwork between general surgery, vascular surgery, radiology, interventional radiology and ICU.

Despite these challenges, most respondents (222;76.3%) expressed interest in participating in a national registry on AMI, demonstrating a strong willingness within the surgical community to collaborate on improving outcomes and organizing the system more effectively.

## Discussion

Acute mesenteric ischemia should be considered not only a complex abdominal emergency, but also a system-dependent condition in which outcomes are strongly influenced by organizational structures, resource availability, and interhospital coordination.

This nationwide survey reveals an important paradox in the current state of AMI care in Spain: while frontline surgeons report high levels of individual competence and experience with complex surgical techniques, systemic infrastructure to support timely diagnosis and treatment is clearly lacking.

Although self-perceived competence does not necessarily equate to optimal clinical performance, the coexistence of high self-reported expertise with significant structural and organizational deficits suggests that system-level factors, rather than individual skills, may represent the primary bottleneck in AMI care. These findings emphasize the need to move beyond individual training and focus on coordinated systems of care, standardized protocols, and multidisciplinary collaboration.

### Lack of institutional protocols

Only 18.9% of surveyed surgeons reported the existence of a formal finalized AMI protocol at their institution (Fig. 1). Several studies confirm that protocolized care increases the use of advanced imaging, reduces time to surgery, and may reduce mortality [10, 15]. Structured pathways allow rapid activation of multidisciplinary teams, mirroring models successfully implemented in stroke care, as Tolonen et al. demonstrated that implementing a structured care bundle for acute arterial mesenteric ischemia significantly reduced mortality in Southern-Finland [9], and their review highlighted key diagnostic and therapeutic strategies, as increased awareness, routine contrast imaging, emergency access to hybrid suites and increased use of endovascular and hybrid revascularisation methods, that can be incorporated into national planning [10, 16]. These examples support the need for similar initiatives across Spanish healthcare regions.

### Unequal access to 24/7 resources

Access to 24/7 interventional radiology (IR) and vascular surgery remains uneven. This is particularly problematic for revascularization, which may include stenting (ante- or retrograde), aspiration thrombectomy, thrombolysis, open embolectomy, patch angioplasty or bypass surgery. Endovascular-first strategies have shown reduced postoperative complications and shorter ICU stays in selected patients [17, 18]and access to hybrid suites enable individualized revascularization strategies with or without a laparotomy [10]. However, these benefits of endovascular options are inaccessible in hospitals lacking 24/7 IR coverage. Guidelines from vascular and emergency surgical societies emphasize the importance of early imaging and coordinated surgical and endovascular decision-making in AMI, underscoring the consequences of these access gaps [2, 3, 19, 20].

Although resource availability may vary according to hospital level, acute mesenteric ischemia is a time-dependent condition that may initially present in hospitals of any level. Therefore, understanding the barriers encountered in different care settings is essential to optimize early recognition, referral pathways, and timely access to specialized treatment. Identifying these challenges may help guide future organizational strategies aimed at improving the management of time-critical abdominal emergencies across diverse healthcare environments.

### Barriers in interhospital transfers

Over 68% of respondents cited difficulty transferring AMI patients to referral centers. Frequent causes include unclear referral pathways and bed unavailability. These logistical challenges have been echoed in recent vascular consensus statements, which call for the creation of regional AMI networks with centralized coordination [2, 10].

### Low awareness of the ‘intestinal stroke’ concept

**Fig. 2:**
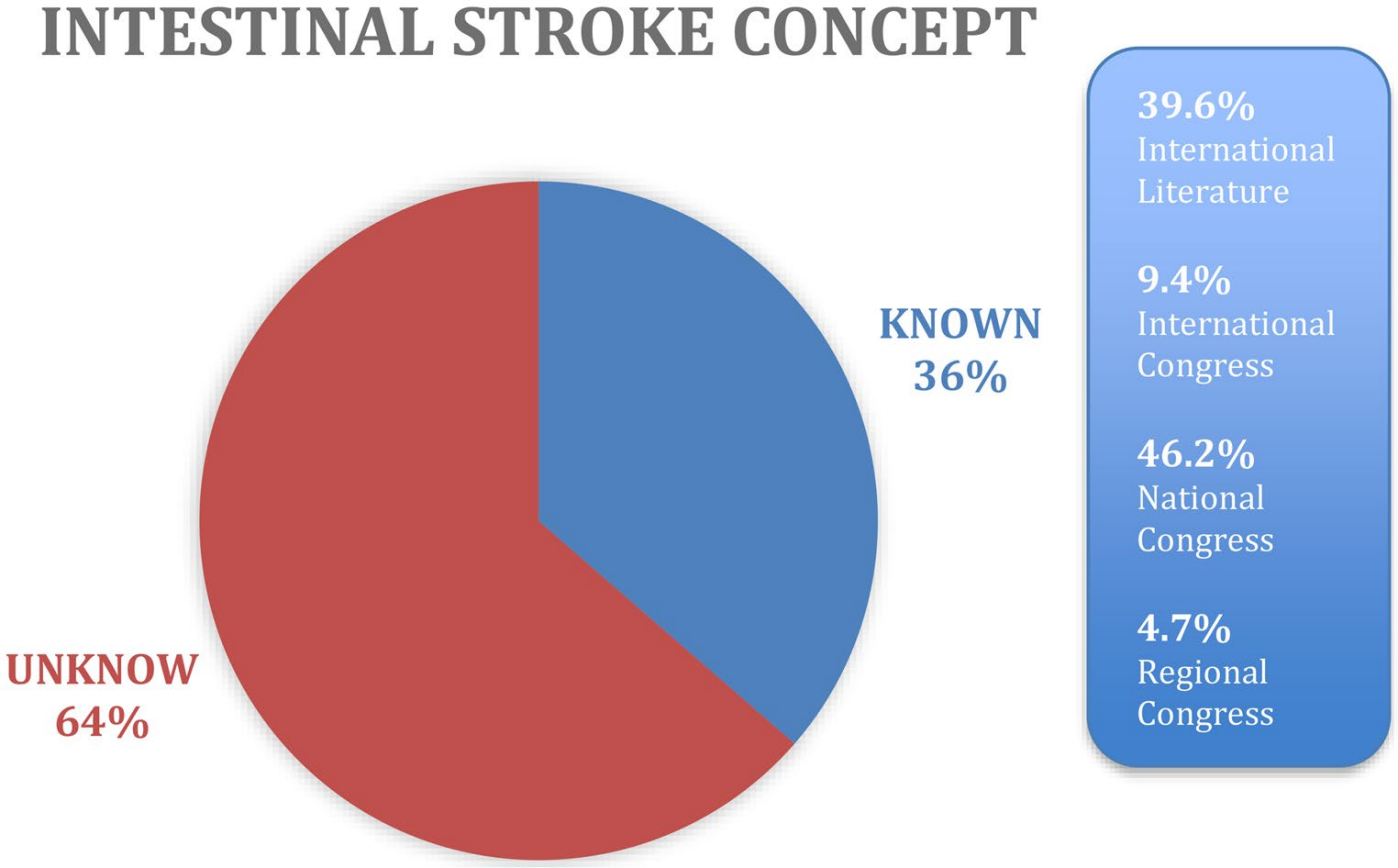
Answers to Have you ever heard of the term “intestinal stroke”? If yes, where?

Despite its proposed use as a communication tool, only 36.4% of surgeons were familiar with the term “intestinal stroke.” (Fig. 2). A similar awareness gap was observed in European Surgical Societies. Incorporating this term into emergency and radiology protocols could increase the perceived urgency and support earlier interventions [9, 10, 21]. The term also aligns with recent literature advocating for biphasic CT as the diagnostic standard to detect early intestinal ischemia [11]. The concept of “intestinal stroke” has been proposed as a communication tool to increase clinical awareness and urgency. Similar to other time-critical vascular emergencies such as ruptured abdominal aortic aneurysm, this conceptual shift may facilitate faster decision-making, prioritization of operating room access, and improved multidisciplinary coordination. The implementation of traffic-light coding systems, as described in the Helsinki experience, has been associated with reduced delays and improved workflow in emergency surgical care [22, 23].

### Engagement of the surgical community

The survey revealed that most surgeons are interested in specialized training and registry participation. This professional readiness is a powerful asset. Previous quality improvement initiatives in gastrointestinal emergencies have succeeded when led by clinician-driven networks [8, 24, 25].

### Regional participation and its implications

**Fig. 3:**
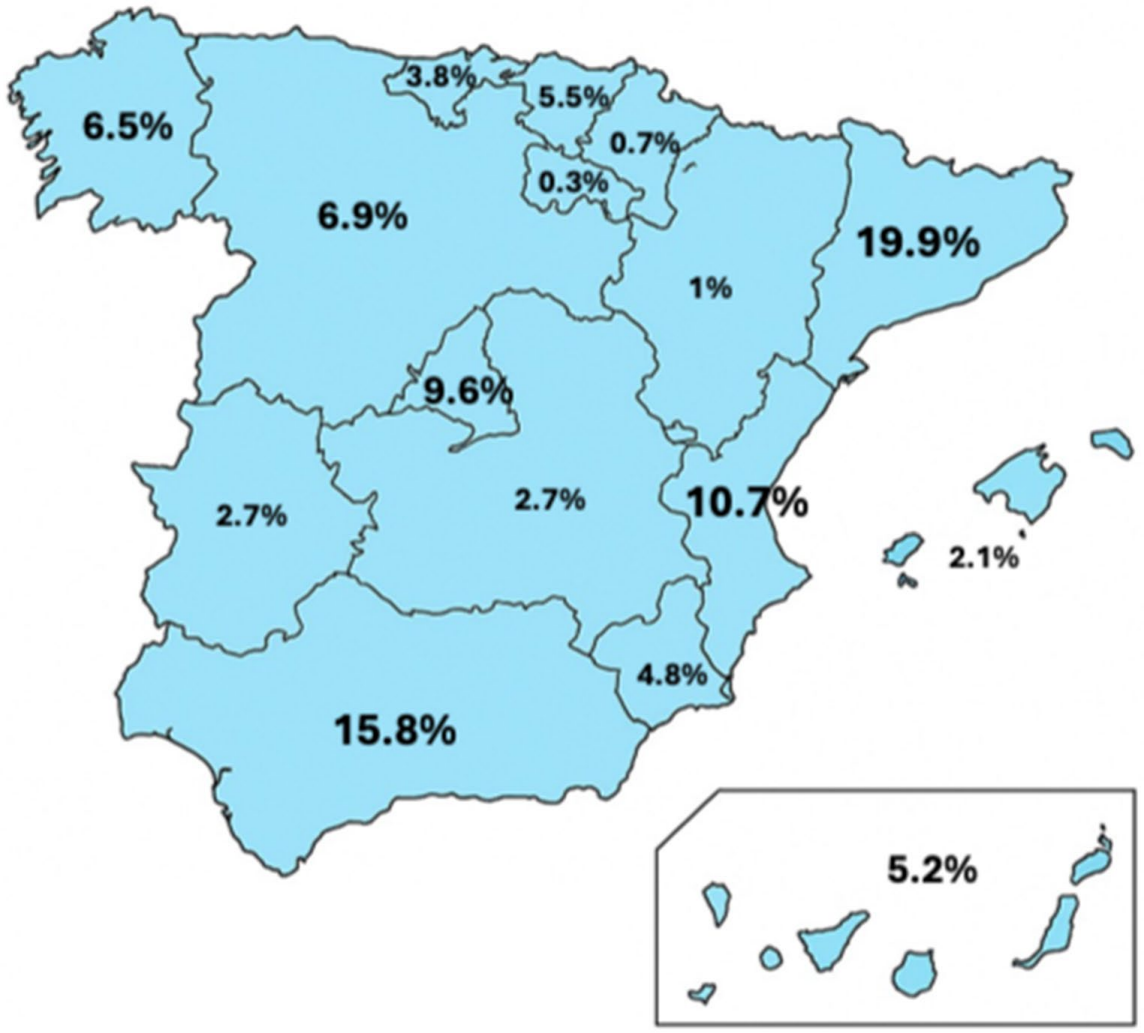
Geographic distribution of survey responses across Spain autonomous communities

This nationwide survey includes respondents from all Spanish autonomous communities, although participation was uneven (Fig. 3). Higher response rates were observed in Catalonia, Andalucia, and the Valencian Community, while some regions like La Rioja and Navarra had minimal representation. This geographic skew may reflect regional differences in healthcare organization, professional networks, or survey dissemination channels.

While this uneven distribution does not invalidate the findings, it introduces a potential selection bias that may limit the generalizability of the results across all Spanish territories. Nonetheless, it highlights an important opportunity: regional disparities in AMI care may also reflect disparities in institutional readiness, awareness, and infrastructure. For example, Catalonia, with its higher engagement in the survey and ongoing development of a coordinated AMI strategy. Limited exposure to advanced endovascular techniques, such as retrograde open mesenteric stenting (ROMS), may further complicate multidisciplinary integration in the management of acute mesenteric ischemia. In addition, variability in training, institutional culture, and regional availability of specialized services may lead to delayed or underutilized involvement of interventional radiology and vascular surgery teams. Early recognition at referring institutions and increased awareness in emergency departments are therefore key factors to ensure timely identification and transfer of patients who may benefit from advanced revascularization strategies [25]. Future national strategies should consider these regional variations in both needs and readiness.

### Qualitative insights reinforcing systemic gaps

The free-text responses offered by survey participants reinforce and deepen the quantitative findings. Numerous surgeons highlighted the lack of standardized care pathways and expressed frustration with the frequent diagnostic delays, especially in emergency departments. Many respondents noted that patient transfers to reference centers were often delayed or denied due to fragmented referral systems, contributing to lost treatment opportunities. A recurring theme was the limited availability of 24/7 interventional radiology and vascular surgery services, described as a critical barrier to implementing effective treatment algorithms. Variability in clinical decision-making based on the experience or preferences of the on-call surgeon was also frequently mentioned. Taken together, these insights emphasize the urgent need for structured protocols, improved interprofessional collaboration, and regionally coordinated systems of care.

### Catalonia as a pilot region

In Catalonia, a multidisciplinary working group-including diagnostic radiologists, interventional radiologists, vascular surgeons, general surgeons and other stakeholders-is developing a standardized regional AMI care pathway for AMI. Drawing inspiration from trauma and stroke models-as well as Helsinki experience-the initiative aims to unify imaging protocols, streamline interhospital referrals, promoting professional training, and enable prospective data collection through registries. While still in development and not yet formally endorsed by health authorities, this collaborative model could serve as a foundation for future national coordination efforts- not only for AMI but also in another time-sensitive abdominal surgical emergencies.

### Study limitations

This study has several limitations. First, although the survey was distributed nationwide, participation was geographically uneven. A few regions—such as Catalonia and Andalucía—contributed a disproportionately high number of responses, while others, including Ceuta or Melilla, were underrepresented. This imbalance introduces potential selection bias and limits the generalizability of findings to the entire Spanish healthcare system.

Second, the survey targeted only general surgeons, excluding key perspectives from other relevant specialties such as Vascular Surgery, Interventional Radiology, Radiology, Emergency Medicine, Anesthesiologists and Intensive Care. However, in many hospitals in Spain, general surgeons are often the first specialists involved in the diagnosis and initial management of acute mesenteric ischemia. Given the multidisciplinary nature of acute mesenteric ischemia, the perspectives of these specialties may provide additional insights into diagnostic pathways, treatment strategies, and organizational barriers. However, this limitation also highlights the need for complementary surveys to capture multidisciplinary perspectives and better inform integrated care pathways. Third, as a self-administered and voluntary instrument, the study is subject to self-selection and social desirability bias. In addition, reported data regarding protocols and 24/7 resource availability were not externally validated, which may affect the objectivity of certain results. Although the survey was distributed to 2,900 surgeons through the mailing list of the Spanish Association of Surgeons, the response rate should be interpreted with caution. Participation depended on surgeons opening the invitation and voluntarily completing the survey, which may introduce self-selection bias.

Finally, the cross-sectional design provides a snapshot in time and does not allow for assessment of temporal trends or causal relationships.

Although this study was conducted in Spain, similar organizational challenges have been reported across different European healthcare systems, suggesting that these findings may be relevant beyond a single national context. Future prospective and interventional studies are warranted to assess the impact of standardized protocols.

## Conclusion

The findings of this survey highlight significant systemic gaps in AMI management across Spain. Despite the availability of advanced diagnostic resources in most hospitals and the high level of experience reported by participants, the absence of standardized protocols, unequal access to specialized services and difficulties in interhospital transfer remain major barriers to optimal patient care. These limitations may contribute to diagnostic delays and worse outcomes in a condition where time is critical. The development of coordinated care pathways and multidisciplinary protocols, such as the initiative currently underway in Catalonia, represents a promising step towards improving management in AMI. Establishing regional networks, promoting professional training and implementing national data registries are essential to reduce variability in care and improve survival rates for this severe and time-sensitive condition.

## Electronic Supplementary Material

Below is the link to the electronic supplementary material.

Supplementary Material 1

## Data Availability

No datasets were generated or analysed during the current study.
