# Supplementary material for: The real-world management of acute mesenteric ischemia in Spain: results from a multicenter national survey
Source: Eur J Trauma Emerg Surg. 2026 May 19;52(1):167. doi: 10.1007/s00068-026-03209-1 (PMC13186878; doi:10.1007/s00068-026-03209-1)
Supplement: Supplementary file 1 — Supplementary Material 1 [file 68_2026_3209_MOESM1_ESM.docx]

## **Appendix 1 – Survey Questionnaire (translated to English)**

**Section A: Demographics**

1. What is your current age?
2. What is your professional rank?
    a. Resident
    b. Attending surgeon
    c. Section chief
    d. Department head
3. What type of hospital do you work in?
    a. Tertiary
    b. Secondary
    c. Community/Comarcal

**Section B: Diagnostic Capabilities**
4. Is multiphasic abdominal CT (baseline/arterial/venous phase) available 24/7?
5. Is a radiology service available 24/7, 7 days a week?

**Section C: Therapeutic Resources**
6. Is interventional radiology available 24/7?
7. Is vascular surgery available 24/7?

**Section D: Institutional Readiness**
8. Does your service have a clinical protocol or guideline for acute mesenteric ischemia (AMI)?
9. Do you know how to manage AMI?

**Section E: Clinical Experience**
10. Have you ever performed an open surgical approach for superior mesenteric artery (SMA) thrombosis?
11. Do you have experience with open abdomen techniques (e.g., temporary abdominal closure, damage control surgery)?
12. Have you ever heard of the term “intestinal stroke”?

**Section F: Systemic Barriers and Attitudes**
13. Do you believe interhospital transfers for AMI patients are too difficult?
14. Would you be interested in participating in a Spanish registry for AMI to improve national data and outcomes?

**Section G: In the free-text section at the end of the survey, you may describe any difficulties you have experienced with this condition and share the experiencies you wish.**
